# Efficacy of scopolamine transdermal patch in children with sialorrhea in a pediatric tertiary care hospital

**DOI:** 10.1186/s12887-020-02336-x

**Published:** 2020-09-17

**Authors:** Majed Al Jeraisy, Maissa AlFuraih, Raghad AlSaif, Bushra AlKhalifah, Hazza AlOtaibi, Mostafa A. Abolfotouh

**Affiliations:** 1 Pharmaceutical Care Department, King Abdullah Specialized Children Hospital, Ministry of National Guard, Riyadh, Saudi Arabia; 2 King Abdullah International Medical Research Center (KAIMRC), Ministry of National Guard-Health Affairs, Riyadh, Saudi Arabia; 3 College of Pharmacy, King Saud Bin Abdulaziz University for Health Sciences Ministry of National Guard-Health Affairs, Riyadh, Saudi Arabia; 4 College of Pharmacy, King Saud University, Riyadh, Saudi Arabia; 5 College of Pharmacy, Qassim University, Qassim, Saudi Arabia

**Keywords:** Drooling, Transdermal, Efficacy, Scopolamine, Hyoscine, Saudi, Neurological disorders, Cerebral palsy, Hospital readmission, ER visits

## Abstract

**Background:**

Drooling is common in children with neurological disorders, but its management is very challenging, Scopolamine transdermal patch (STP) appears to be useful in controlling drooling, although it is not approved for this indication and there are limited clinical studies about its effectiveness. This study aimed (1) to assess the impact of STP use on the severity of drooling and on the frequency of emergency department (ED) and hospital readmission (RA) visits related to drooling, and (2) to determine the level of family satisfaction with STP when used in children with neurological disorders.

**Methods:**

This is a retrospective cohort study of all pediatric patients aged 3–14 years, with non-progressive neurodevelopmental disability, who used STP for more than one year during the period between April 2015 and July 2018 (*n* = 44). Data on demographics, clinical status, comorbidities, STP dose and duration, other medications, ED and RA visits were collected. Follow-up phone-call interviews with parents/caregivers were performed using a parent-reported frequency and severity rating scale of sialorrhea. Absolute and relative risk reductions were calculated to assess the impact of STP on ED and RA visits. Significance was considered at *p*-value of ≤ 0.05.

**Results:**

STP use showed significant reduction in severity of drooling (*p* < 0.001), wiping of the child’s mouth (*p* < 0.001), bibs or clothing changes (*p* < 0.001), choking and aspiration of saliva (*p* = 0.001). The Relative Risk Reduction of the drooling-related ED and RA visits were 86% and 67% respectively. Nearly two-thirds (60%) of caregivers were satisfied with using STP.

**Conclusions:**

This is the first study of its kind done in Saudi Arabia demonstrating favorable impact of STP use by children on the consequences associated with drooling and with the frequency of ER and RA visits due to drooling. Development of a medication use protocol is recommended to standardize STP treatment in order to optimize its effectiveness. This study serves as baseline information for future prospective interventional studies.

## Background

Sialorrhea (drooling) is a biological condition characterized by salivary incontinence or the automatic spillage of saliva over the lower lip. The average saliva secretion in healthy individual is 0.5–1.5 L/day [1]. Sialorrhea is abnormal over the age of about 4 years [2], and is often found in children with neurological disorders. It could be due to failure to retain saliva inside the mouth, or difficulty with swallowing [3]. It was prevalent in 40% of a population-based study of 7 to 14-year-old children with cerebral palsy (CP) [4], and in 58% of children with CP attending special schools in the UK [5], while the pooled prevalence estimate determined in a recent meta-analysis was 44% [6]. Sialorrhea has a negative impact on functional and clinical outcomes for patients, families, and caregivers.

Patients with sialorrhea will manifest this problem in one of two principle ways. Anterior sialorrhea is when patients have excessive anterior or forward spillage of saliva from their mouths onto their faces and clothes, causing difficulty with cleanliness, skin care, and socialization [7–9]. Posterior sialorrhea occurs when the trigger to swallow is impaired or missing. These patients have excessive posterior spillage of saliva into the hypopharynx, and pooled saliva may lead to congested breathing, coughing, gagging, vomiting, and at times aspiration into the trachea [10]. Unrecognized and silent pneumonia can occur [11].

Reported treatment options have included behavioral modification therapy, oral or topical anticholinergic medications, surgical excision of salivary glands or duct relocation, and chemodenervation with botulinum toxin [12]. Pharmacological medications such as glycopyrrolate, an anticholinergic agent, decrease saliva secretion through the parasympathetic autonomic nervous system [13]. Glycopyrrolate is approved by the US Food and Drug Administration (USFDA) for drooling in pediatric patients with neurologic conditions [14]. Scopolamine is an anticholinergic agent with antiemetic and hypnotic-sedative properties. As scopolamine blocks parasympathetic innervation of the salivary glands, one of its indications is to reduce saliva secretion [15].

Scopolamine transdermal patch (STP) has been used to decrease salivation in adult patients and was reported in 1984 by Dettman et al. [16] and Gordon et al. in 1985 [17] with a significant reduction of the salivary flow. A double-blind, placebo-controlled study in 1994 [18] on ten developmentally delayed children supported earlier reports of the safety and efficacy of STP for reducing excessive drooling. Another study in Nijmegen, 2006 [19] conducted in an out-patient clinic on 45 pediatric patients with CP and severe drooling, using scopolamine patch and botulinum toxin, showed that both scopolamine and botulinum toxin significantly decreased salivary flow. In 2010, a prospective randomized double-blind, crossover, placebo-controlled clinical trial [20] done in Spain for 30 patients with severe disabilities came up with significant reduction in drooling. A multi-center randomized controlled trial was initiated to identify whether glycopyrronium or scopolamine was more effective in treating drooling in children with non-progressive neurodisability, and showed that scopolamine and glycopyrrolate were both clinically effective in treating drooling, but scopolamine produced more problematic side effects leading to a greater chance of treatment cessation [21]. In a recent study by Reid et al., [22] benzhexol, glycopyrrolate, and scopolamine reduced drooling, but improvement was offset by adverse side effects, glycopyrrolate performed best. A number of side effects have been reported with STP such as; blurred vision, sedation, dry mouth, drowsiness, dizziness, increased seizure frequency, constipation, urinary retention, difficulties in hot weather due to decreased sweating and mild itching/redness at the application site [21, 23, 24].

Despite the abundance of reports on the efficacy and safety profiles of each treatment option, definitive conclusions are difficult to draw, given the heterogeneous nature of the patient populations studied and the different outcome measures used in the various studies [12]. The aims of this study were: (1) to assess the impact of scopolamine transdermal patch use on the severity and frequency of drooling, (2) to assess its efficacy in pediatric patients with regards to the frequency of drooling-related emergency department (ED) visits and hospital readmission (RA) visits, and (3) to determine the level of satisfaction of families and/or caregivers and their convenience before and after using this medication.

## Methods

### Study design

This was a retrospective cohort study among pediatric patients aged 3 to 14 years, with non-progressive neurodevelopmental disability, using STP for more than one year during the period between April 2015 and July 2018.

### Study setting

This study was conducted at King Abdullah Specialized Children’s Hospital (KASCH), a tertiary pediatric hospital in Riyadh, Saudi Arabia, with a total capacity of 552 beds. STP is a non-formulary and restricted medication in the hospital, and there are no hospital guidelines or protocol to standardize its use. The usual hospital practice of managing patients with excessive salivation is to start them on glycopyrrolate for a week, if there is no response [non-response was defined as no change in the frequency of salivation as mentioned by the child’s parent/caregiver], then to switch to STP, starting with ¼ patches (0.375 mg) then gradually increased to full patches (1.5 mg), or to the maximum tolerated dose of their medication. STPs were placed on cleaned skin behind the ear once daily. The backing of the patch was covered to expose the prescribed portion of it, and an occlusive dressing was then applied over the patch as per usual practice [3].

### Study subjects

This study included all pediatric patients aged 3 to 14 years, with non-progressive neurodevelopmental disability, who started on glycopyrrolate then shifted to STP for a minimum of one year during the period between April 2015 – the date of launching a new electronic medical record (EMR) system in the hospital- and July 2018, and who were still alive during the study period (*n* = 44). This period was chosen so as to allow for more accurate data to be collected from the EMR about the management of drooling. Of the records of 69 children, those with progressive neurodevelopmental disability, those whose parents could not be reached for interview, and those who were on STP for less than one year at the time of study were all excluded. All patients who were on dual therapy (glycopyrrolate + Scopolamine) were also excluded.

### Data collection

The following data were retrieved from electronic medical records of all children who were on STP: Demographic characteristics and clinical data, frequency of ED and RA visits, comorbidities and dose of STP and its duration. The severity of drooling was determined subjectively using a parent-reported frequency and severity rating scale of sialorrhea [25, 26]. Follow-up interviews with parents/caregivers of the patients were performed, one year or more after commencing the patches, either by a phone call for the outpatients (*n* = 38) or face-to-face interview if the patient was hospitalized (*n* = 06). The questionnaire was composed of 5-point scaled questions, to assess the frequency of different consequences associated with drooling (1-none, 2-mild, 3-moderate, 4-severe, and 5-Very severe) [26]. Parent/caregiver was asked one more question to assess their satisfaction with the use of STP (1-Not satisfied, 2-Less satisfied, 3-Neutral, 4-Satisfied and 5-Very satisfied). There was an independent witness from outside the research team witnessing the consent process with parents over the phone, with their agreement to participate in the interview and allow for their children’s recorded data be used in research. The study was approved by the Institutional Review Board (IRB) of Ministry of National Guard-Health Affairs, Riyadh, Saudi Arabia,[*Ref.#RSS18/008/R*].

### Data analysis:

The data was analyzed using SPSS (Version 25). Descriptive statistics such as; percentage, mean ,standard deviations (SD), median and interquartile range (IQR) were used. To compare between the mean score of consequences on the child due to drooling before and after STP use, Wilcoxon signed-ranks test was used. The relative risk reduction (RRR) and the absolute risk reduction (ARR) and their corresponding 95% Confidence intervals, and the relative risks (RR) were all calculated, to assess the impact of using STP on the frequency of ED visits and hospital admission visits related to drooling. There was no missing data. Significance was considered at *p*-value of < 0.05.

## Results

Records of 69 patients were reviewed, 44 (63.8%) met the inclusion criteria. Children with progressive neurodevelopmental disability (*n* = 19), those whose parents could not be reached for interview (*n* = 04), and those who were on STP for less than one year at the time of study (*n* = 02) were all excluded. Patients manifested sialorrhea in one of two forms; posterior drooling (*n* = 29, 66%) and anterior drooling (*n* = 15, 34%). Demographics of the included patients are presented in Table 1. Male/female ratio was 1:1.1, with a median age of 93 months (IQR = 64), median height of 105 cm (IQR = 32) and median weight of 16.8 kg. (IQR = 15.9). Comorbidities included epilepsy (79.5%), global developmental delay (61.4%) and gastro-esophageal reflux disease (59.1%). With regard to scopolamine adverse effects, one-third of all patients (36.4%) had tachycardia, 13.6% had visual disturbance, and 9.1% had urinary retention. Dry mouth, skin irritation or drowsiness were not among the side effects mentioned by parents/caregivers. A total of 29 children were receiving regular suction, and 60% of their parents/caregivers reported decrease in suction after using STP.

**Table 1:** Personal and disease characteristics of children with Sialhorrea

Variable	*N* = 44
***Patient’s characteristics***	
Sex (M/F)	23/21 (1.1:1)
Age in months [Md & IQR]	93.0 (64)
Height in cm. [Md & IQR]	105 (32)
Weight in kg. [Md & IQR]	16.8 (15.9)
***Comorbidities***^***a***^	
Epilepsy [n, %]	(35) 79.5
GDD [n, %]	(27) 61.4
GERD[n, %]	(26) 59.5
***Side effects of STP use***^***a***^	
Eye problems [n, %]	(6) 13.6
Urinary retention [n, %]	(4) 9.1
Tachycardia [n, %]	(16) 36.4

^a^ figures for this variable are not mutually exclusive, *GERD *Gastrointestinal reflux disease, *GDD *Global developmental delay, *Md *Median, *IQR *Interquartile range, *STP *Scopolamine transdermal patch

**Fig. 1:**
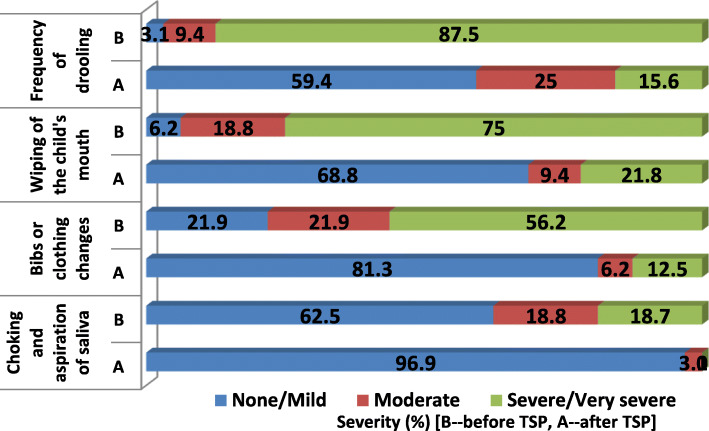
Severity of consequences associated with sialorrhea before and after scopolamine STP use

Table 2; Fig. 1 show the distribution of children with neurological disorders according to the frequency (mean score ± SD) and severity of different consequences associated with drooling before and after STP use. The proportion of children with severe/very severe drooling was reduced significantly from 87.5% of children before STP use to 15.6% after STP use (*p* < 0.001). Severe/very severe need for wiping of the child’s mouth was prevalent in 75% of children before STP use, and it was reduced significantly to 21.8% after STP use (*p* < 0.001). Severe/very severe need for clothing changes were prevalent in 56.2% and 12.5% of all children before and after STP use, respectively (*p* < 0.001). Choking and aspiration of saliva was severe/very severe in 18.7% of children before STP use, while none of the children suffered from this severe choking after STP use (*p* = 0.001). Parents and/or caregivers of the majority of children with sialorrhea (59.4%) were satisfied/very satisfied with STP use by the children (Table 2).

**Table 2:** Consequences associated with drooling before and after scopolamine skin patch and family satisfaction among children with drooling

	Scopolamine STP use	Severity of Sialorrhea							Statistical significance^a^
		*None*	*Mild*	*Moderate*	*Severe*	*Very severe*	*Severity score*		
***Consequences associated with drooling***		%	%	%	%	%	Mean	SD	*P*-value
Frequency of drooling	*Before*	0.0	3.1	9.4	31.3	56.2	4.41	0.80	*P*<0.001
	*After*	28.1	31.3	25	9.4	6.2	2.34	1.18	
Frequency of wiping of the child’s mouth	*Before*	3.1	3.1	18.8	9.4	65.6	4.31	1.09	*P*<0.001
	*After*	31.3	37.5	9.4	15.6	6.2	2.28	1.25	
Frequency of bibs or clothing changes	*Before*	9.4	12.5	21.9	28.1	28.1	3.53	1.29	*P*<0.001
	*After*	46.9	34.4	6.2	9.4	3.1	1.88	1.10	
Choking and aspiration of saliva	*Before*	56.3	6.2	18.8	12.5	6.2	2.06	1.37	*P*=0.001
	*After*	84.4	12.5	3.1	0.0	0.0	1.19	0.47	
***Family satisfaction***	*Unsatisfied* *%*		*Less satisfied* *%*		*Neutral* *%*		*Satisfied* *%*		*Very satisfied* *%*
	12.5		6.2		21.9		12.5		46.9

^a^Wilcoxon signed-ranks test was applied

Table 3 shows the ED and the RA visits of children due to drooling before and after STP use. The rate of ED visits due to drooling dropped from 33% before STP use to 5.4% after, with an ARR of ED visits of 27.6% (95% CI: 9.4% − 45.6%, *p* < 0.001), and RRR of 86%. Relative risk was 0.16 reflecting a reduction of the rate of ED visits by 84% after STP use. With regard to RA of children due to drooling, the rate of RA dropped from 44.4% before STP use to only 8.7% after, with an ARR of 35.7% (95% CI: 15.7% – 55.7%, *p* < 0.001), and RRR of 67.1%. Relative risk was 0.20, reflecting 80% reduction in RA rate after STP use.

**Table 3:** Risk reduction on emergency department (ED) visits and hospital readmission (RA) visits due to scopolamine patch use among children with drooling

***Emergency department visits (ED):***	
Rate of ED visits due to drooling before scopolamine patch use (CER) %	33.0
Rate of ED visits due to drooling after scopolamine patch use (EER) %	5.4
ARR on ED visits %= CER - EER = 33.0–5.4	27.6 (95%CI:9.4–45.6, *p* < 0.001))
RRR on ER visits % = (CER – EER)/CER = (33.0–5.4)/33.0	86.0
Relative risk (RR) = EER/CER = 5.4/33.0	0.16
***Readmission visits (RA):***	
Rate of RA visits due to drooling before scopolamine patch use (CER) %	44.4
Rate of RA visits due to drooling after scopolamine patch use (EER) %	8.7
ARR on RA visits %= CER - EER = 44.4–8.7	35.7(95% CI: 15.7–55.7, *p* < 0.001)
RRR on RA visits %= (CER – EER)/CER = (44.4–8.7)/44.4	67.1
Relative risk (RR) = EER/CER = 8.7/44.4	0.20

*CER *Control event rate, *EER *Experimental event rate, *RR *Relative risk, *ARR *Absolute risk reduction, *RRR *Relative risk reduction

## Discussion

A number of studies have detected a reduction in saliva secretion with STP treatment among children with neurodevelopmental disorders, yet efficacy varied between patients [25, 27–29]. Our study showed favorable impacts of STP use by children on the consequences associated with drooling, with a significant reduction in the proportion of children with severe/very severe drooling, need for wiping of the child’s mouth, need for clothing changes and choking. The results of the present study were consistent with those published by other authors such as Mato et al. [20] who studied 15 patients with mental retardation, where treatment with scopolamine led to a significant reduction in drooling at 24, 48, and 72 h after application of the skin patch, compared with placebo. Our findings were also in agreement with a previous study [25] reporting that drooling completely resolved in one third of cases in a group of 11 children with mental retardation and moderate-severe drooling. However, this medication should be used with caution, considering the side effects shown in the present study, where more than one third of all patients suffered tachycardia. Moreover, around 80% of patients presented with epilepsy as a comorbidity, and one of the side effects of scopolamine is that it could interact with anti-epilepsy medication and that it could increase seizures.

Recently the DRI trial of Parr et al. [21] showed that scopolamine and glycopyrrolate were both clinically effective in treating drooling, but scopolamine produced more problematic side effects leading to a greater chance of treatment cessation. The timing of drug administration may explain this discrepancy between the results of the trial and those of our study, where in Parr’s trial children were randomly assigned to these two medications, at the same time, while in our study, the situation is different, as the aim was to assess retrospectively the STP outcome among only those who had not shown a positive response to glycopyrrolate. This means that our study reflected the STP treatment outcome only among those who did not respond positively to glycopyrrolate, and did not investigate the glycopyrrolate treatment outcome for those who had responded to it positively. Meanwhile, the outcome of scopolamine in our study could be confounded by some factors such as; the type of drooling [anterior and posterior], scopolamine dosage, duration of treatment, and previous medications. Thus, a large scale prospective study is necessary to clarify this point in our setting.

The difficulty for quantifying drooling in patients with disabilities may explain the variability of results, with efficacies between 19% and 67% being reported in the literature [25, 28, 29]. In the present study, we relied upon more objective measures of impact of STP use on posterior drooling, which is the impact on the frequency of ED and RA visits related to drooling. In our study, the RRR of the drooling-related ED and RA visits were 86% and 67% respectively, with 84% and 67.1% reductions in ED and RA visits respectively. This may explain the favorable impact on the quality of life of the children under scopolamine use as well as satisfaction of their families of its use.

### Strengths and limitations

The main strength of this study is that it is the first in Saudi Arabia demonstrating favorable impact of STP use in drooling among children with neurological disorders. With regards to its limitations, the severity of drooling was determined subjectively using their parents/caregivers’ frequency and severity rating scales of sialorrhea, with a possible recall bias. The use of different methods of interview [face to face and phone calls] might add more possible information bias. A potential limitation was the inclusion of patients who were using STP for more than one year, and these were the patients who had a more positive effect of the medication and less side effects than those who only used STP for shorter period of time, and stopped its use probably due to its side effects or insufficient effect. This may lead to an increase in the favorable impact of STP use, and influence the conclusion of the study.

## Conclusions

To our knowledge, there are no similar studies conducted in Saudi Arabia to assess the effectiveness and safety of STP in pediatric patients. The results showed that, after using STP, there was significant reduction in the severity of drooling, wiping of the child’s mouth, bibs or clothing changes, choking and aspiration of saliva, reduction in the rate of ED and RA visits, and favorable level of family satisfaction with medication. Our findings were consistent with previous studies. This study could serve as baseline information for future prospective interventional studies on the appropriate timing, duration and dosage of STP treatment for pediatric patients with drooling.

## Data Availability

Most of the data supporting our findings is contained within the manuscript, and all others, excluding identifying/confidential respondent data, will be shared upon request.

## Abbreviations

KAMC: King Abdulaziz Medical city
MNG-HA: Ministry of National Guard-Health Affairs
KASCH: King Abdullah Specialized Children’s Hospital
IRB: Institutional Review Board
STP: Scopolamine transdermalpatch
ED: Emergency department
RA: Readmission
ARR: Absolute risk reduction
RRR: Relative risk reduction
RR: Relative risk
USFDA: US food and drugadministration
